# Two epochal turns of inequality, their significance, and their dynamics

**DOI:** 10.1186/s40711-021-00143-0

**Published:** 2021-03-22

**Authors:** Göran Therborn

**Affiliations:** The Old Schools, University of Cambridge, Trinity Lane, Cambridge, CB2 1TN UK

**Keywords:** Inequality, De-industrialization, Post-industrial society, Poverty, Financialization

## Abstract

At the end of the twentieth century, two historical turns of economic inequality happened. Among the developed countries of the Global North, the secular trend of decreasing intra-national inequality turned into its opposite. At about the same time, the long period of global inequality began to bend down, among households as well as among nations, a turn less noticed but more significant than the reduction of extreme poverty in the South. The foundation of the former turn was the beginning of de-industrialization in the North, and the coming of a post-industrial society, very different from the one predicted. The paper analyzes the trigger of the turn and the central dynamics of the new inequality in the rich North, financialization, and the digital revolution. It then tries to answer two questions about the global turn: Was the decline of global inequality causally connected to the increase of Northern intra-national inequality? Will there be a development of industrial societies in the South? The answer to both is no. What lies ahead is more likely a global convergence of intra-national unequalization, albeit with both different and similar dynamics, as the decline of extreme poverty in the South is leading to inequality increases comparable to those of the North. Post-industrialism has no egalitarian dialectic like that of industrial capitalism, but the dynamics of the twenty-first century inequality are likely to be confronted not only with popular protest movements but also with an emergent scholarly and intellectual Egalitarian Enlightenment.

## When history turned

The social landscape of human (in)equality in the early twenty-first century was shaped by major historical turns in the last decades of the previous century. Economic inequality in the most developed parts of the world took a U-turn in the 1980s, from a secular direction towards declining inequality, in particular after World War II, to a path of mounting inequality. With striking simultaneity, international inequality also changed direction. It had been increasing since the Western industrial revolution and imperialist expansion into Asia. With decolonization after World War II, which meant that the Chinese and Indian economies could start to grow again, international inequality stopped accelerating and slowed down on a high plateau. In the late 1980s, the international inequality curve began to bend down. After 2000, global inequality among households also started the descent.

The two other fundamental dimensions of inequality, vital inequality of health and life expectancy, and existential inequality of recognition/disregard, autonomy/heteronomy, and respect/humiliation, also moved in new directions in the last third of the past century, although less dramatically (UNDP 2019). Unequal life expectancy in the world diminished, but the unevenness, precarity, and reversibility of vital progress were highlighted by the havoc wrought by HIV/AIDS and by the restoration of capitalism in the former Soviet Union, which suddenly sank life expectancy by up to 10 years in South Africa and for Russian males by 7 years, respectively. In Western Europe and North America, a class gap of longevity opened up and started to widen.^1^ These new inegalitarian life tendencies foreboded a century starting with the lethal opioid epidemic in the USA, Ebola outbreaks in Africa, and several virus epidemics up to the ongoing COVID-19 (Coronavirus Disease 2019) pandemic.

With respect to existential inequality, on the other hand, landmarks of equalization were achieved, if far from full human equality. In the USA, African-Americans finally got the right to vote, and the last explicitly racist state, apartheid South Africa, imploded. Patriarchal family legislation was abolished in most parts of the world (Therborn 2004:chs. 2:3, and 3), and women got entry into public space and public office. Current movements and struggles around racism and sexism are taking place on a field laid out by the abovementioned landmarks.

This paper is concerned with understanding the two historical turns of economic inequality at the end of the twentieth century and their implications and prospects for the twenty-first century. It aims at historically situating and explaining the turn to increasing inequality in the Global North from around 1980 and at identifying the major drives of the continuing process of unequalization. It will try to answer whether there is a connection between the turns to increasing intra-national inequality in the North and descending international global inequality. It aims at spelling out the socio-historical significance of the rise of the Global South. Is it the beginning of the end of human poverty? Will industrial society relocate to the South? Or is it more significantly a beginning of convergence with the North in similar tendencies of intra-national inequality as well as in international standing? Currently, the world, North and South, is moving towards ever more economic inequality. This is a socio-politically unstable process, even if there is no longer any powerful dialectic undermining it, as there was in industrial capitalism. The paper ends by indicating an emergent Egalitarian Enlightenment, which might add to the instability of ongoing inegalitarian tendencies.

## The inegalitarian turn in the Global North

In 2008, the OECD (Organization for Economic Co-operation and Development), the economic cooperation organization of the most developed countries, took note of a new phenomenon, which had evolved over the past two decades, the rise of economic inequality. Analysis and concern continued in a series of reports (OECD 2008, 2011, 2015, 2018, 2019). What had happened constituted an epochal shift of income development, a turn away from a general post-World War II tendency of economic equalization in the OECD world of the Global North, indeed from the main distributive trend of the area since World War I (Atkinson and Piketty 2010:692-3). It was a major surprise to mainstream economic wisdom, which held that after rising in the developmental phase of transition from agricultural to the industrial economy, inequality would decline with industrial development, the famous Kuznets curve of an inverted U (Kuznets 1955).

There is a vast literature on this turn and its continued dynamics, much of it referenced in the long OECD reports mentioned above. Technological change favoring high skills, and “globalization,” mainly of finance and labor markets after trade having been found of little or no systematic impact, have been the two favorite variables. This paper starts from finding two significant lacunae in the multivariate approaches. First, they pay no or scant attention to the large-scale turn of socio-economic history involved, to the egalitarian potentialities of industrial societies, to the significance therefore of Northern de-industrialization and arrival of post-industrial societies, and to the importance for inequality of industrial prospects in the Global South. Second, they do not take into account the abruptness of a large-scale socio-economic change, which calls for politico-economic historical attention to triggers.

While epochal, the turn to inequality in the developed North was uneven and unequal (OECD 2008:27), with counterbalances of variable weight. From a historical perspective from 1900 to the second decade of the twenty-first century, the main patterns of change may be identified between two poles visualizable as a U and a reversed J. The USA, with the whole Anglosphere and the Nordic countries of Sweden and others, has trajectories of a U-shape, while, e.g., France, Germany, and Japan more resemble a reversed J.^2^ The political economy analysis of triggers of the turn in this paper will focus on the USA, the decisive pivot of the turn, and Sweden, its unexpected continental European pioneer. The simultaneity and similarity of the turn of the two social poles of the OECD area, of forceful market liberalism and comprehensive social democracy, provide an edge to the analysis of the forces involved, while being unrepresentative of the self-constrained reversed J option.

The inegalitarian U-turn of the Global North around 1980 was a seismic social shift, which cannot be understood or explained by single variables. The major foundation of modern egalitarian policies and rights, developed industrialism, was suddenly trembling and breaking up. The end of the industrial mold of capitalism, and therewith of the conception of a developed society as an industrial society, is the most profound and fundamental cause of contemporary inequality in the rich North.

Industrial employment reached its peak in the developed countries in 1965–1973, a downward slope began in the USA and a few other countries in the late 1960s and then accelerated downwards with the mid-1970s oil crisis. De-industrialization has reshaped the social landscape (Table 1).

**Table 1:** Industrial employment (OECD definition, including manufacturing, construction, mining, and utilities) in some developed countries, from peak to 2018 (percent of total employment)

Country	Industrial peak	2018
France	1973—40	20
Germany	1970—49 (West Germany)	25
Japan	1973—37	26
Sweden	1965—44	21
UK	1911—52	18
USA	1967—36	18

Sources: peak figures: Therborn (1995:69); 2018: ILO (2019)

Successful, socially molding industrialization, capitalist as well as socialist, provided a basis for a broad, relatively egalitarian society, after usually horrific primitive industrial accumulation. It brought non-elite working people together, creating large working-class communities that developed their own, largely egalitarian culture and organizations, thereby promoting social and political strength. Under capitalism, this was the class dialectic Marx indicated and predicted in the mid-nineteenth century. Industrial capitalism would develop its adversary, the working-class whose livelihood was a cost to capital in size, cohesion, and strength. This led to political democracy, for which the labor movement was the main force (Therborn 2020a) to trade union-based workers’ rights and welfare state social rights. Capital income shares fell, manual wages increased and narrowed their distance to the salaries of managers and white-collar employees (Lindert and Williamson 2016:200ff). That is the story of the “Great Leveling” of the twentieth-century industrial world of 1945–1975.

Industrial technology had a particular pro-equality potential, which could be realized in socio-politically favorable contexts. Its perhaps most typical twentieth-century component was the assembly line. It ensured the high productivity of unskilled workers, which in a positive context implied compatibility between profitability and high wages for masses of workers. The US auto industry with its well-paid and, after successful ferocious fights, unionized and socially insured workers summed up the pro-worker potential of industrial technology, from the 1949 landmark collective agreement referred to as the “Detroit Consensus.”

Developed industrialism meant a norm of stable employment at living wages, workers’ rights, and civic rights, doing away with casual labor. This led to the standardization and stabilization of sexual and family relations after the disruptions of nineteenth- and early twentieth-century industrial urbanization with plummeting marriage rates and soaring numbers of extra-marital children. The family of developed industrialism saw a peak of marriage in Europe and North America, where the universal marriage was not a modern norm, unlike in Asia and Africa.^3^ It was a male-dominated nuclear family, with a historical record proportion of female housewives. However, it provided a relatively equal launching-pad for social mobility, with supply routes opened by a broadening of the educational system and increasing demand for educated white-collar labor from rapidly growing offices of business and public services.

Under post-industrialism, these conditions of developed industrial societies are all being reversed, with fracturing and inegalitarian consequences. Post-industrial capital strives to distance and divest itself as much as possible from productive work, outsourcing, and subcontracting it instead. With digital platforms, in the so-called gig economy, it tries to profit from other people’s work without employing them. Fragmentation and dispersion of the class of workers are normal working conditions in the service sector. The low skill-high productivity jobs of the developed industry have decreased dramatically, and there is no equivalent service technology in sight. More than half of all new jobs in the OECD in 1995–2007 were “non-standard jobs,”—temporary, part-time, or own account—amounting to almost a third of total OECD employment in 2013 (OECD 2015:29).

## Post-industrial societies and the new power of capital

The classical work on the transition from industrial to post-industrial society is Bell’s *The Coming of Postindustrial Society* (1973/1976/2003), a bold and wide-ranging book. In relation to issues of (in)equality, it is disappointing and wrong, however. Disappointing not only because it has virtually nothing to say about the issue, but mainly because from his lofty Brahmin perspective, Bell did not see the egalitarian working-class communities, the characteristic of historical industrial societies, and their beginning disintegration and destruction into a “Rustbelt” with the coming of post-industrialism. Bell was fatally wrong in his forecast of post-industrial capitalism, envisaging the coming society primarily as technocratic and science-based, in which capitalists would be increasingly marginalized. Financial capital was a “strategic resource” of industrial society, and to be replaced by “human capital” (p. lxxxv), there would be an “enormous growth in the ‘third sector,’ the non-profit area outside of business and government” (p. 269). “And as ownership of companies becomes disbursed through mutual funds and pension plans, we approach the anomaly of a capitalist system without capitalists” (p. lxv). The “dominant figures” of power, succeeding businessmen, will be “Scientists, Research men” (p. 359). Bell’s basic ideas of a post-industrial society developed in the early 1960s (p. ciii) and have the imprint of the scientism and managerialism of that time, before the turbulence of the late 1960s and before the crises of the 1970s, indicating a conceivable evolutionary path to a post-industrial society if the later upheavals had never happened.

Post-industrialism did not come by scientists and research men, but by capitalists. Their path was traced by two economists without elaborating its destination, Barry Bluestone and Bennett Harrison—*The Deindustrialization of America* (Bluestone and Harrison 1982) and *The Great U-Turn* (Harrison and Bluestone 1988), little noticed by the mainstream literature. The post-industrial society of the Global North arrived by the sharp fall of the rate of profit in the 1970s, particularly in the then central and leading manufacturing sector, and with the concerted efforts of capital and pro-capital governments, including the Social Democratic Government of Sweden from 1982,^4^ to restore it. This was a cross-national phenomenon, although of varying severity. Many factors contributed to the profit squeeze, including saturation of domestic markets for consumer durables, newcomers’ export drives, by recovered Germany and Japan returning to the world market, and by emergent East Asian economies, for example, South Korea, and Singapore, and cost increases from the oil price hike and trade union strength. The late 1960s to 1970s was the summit of working-class strength and ambition, in unionization, in successful strikes (e.g., bringing down the British Conservative government in 1974), and in bold political projects (e.g., the socializing so-called Meidner funds in Sweden, and a “rupture” with capitalism program in France of the Common Front). In the USA, there were the War on Poverty, the Great Society, the civil rights, finally bringing democracy to the US South, and the largest reduction of economic inequality in the post-war USA (Kelly 2009:160). Fear of organized labor was one of the vehicles of Northern post-industrialism.

The solutions adopted had some underpinning in technological evolution. Manufacturing employment had started to decline slightly before the crisis in some countries (e.g., USA and Sweden), and Swedish and other attempts at responding by state-supported reindustrializing policy failed. The parameters of international competition had changed, the increasing productivity of industrial technology needed less labor for producing goods in demand, and scientific developments opened up novel technical and economic applications. Nevertheless, like the previous shift from agriculture to industry, the coming of post-industrialism should not be assumed to be a smooth advance of economic progress. It arrived through struggles for power and gain in contingencies of history.

The international context of the 1970s to early 1980s was turbulent. The post-war system of currency stability had broken down, the oil cartel OPEC (Organization of the Petroleum Exporting Countries) had emerged as a new power player, the sudden flush of petrodollars meant a new investment landscape, and the Keynesian economic policy consensus had blown up, in front of simultaneous inflation and mass unemployment. In this context of pressure and turmoil, the forces of capital, managerial, political, “scientific,” and ideological mounted a large-scale counter-offensive, which turned out largely successful, particularly in the light of failed attempts at industrial revival. Profitability was restored, while the decline of manufacturing employment accelerated in the USA, particularly in 2000–2010, when it lost a third of its total (Acemoglu et al. 2014). From a post-war low of 6.5% in 1982, the US net corporate profit rate went up to almost 9% in 1985, continuing to 11% in 1995, and through a bumpy ride to 13.3% in 2013. The share of corporate profits in national income jumped to a post-war record in 2016 (Wolf 2017:27f). Between 1973 and 1986, the number of production and non-supervisory workers in US manufacturing declined by 1.75 million while all other economic sectors hired more workers. Hourly earnings fell for all workers except miners (Harrison and Bluestone 1988:297). The de-industrializing restoration of the profit rate in the USA meant another historical break, a decoupling of employee compensation from labor productivity. From 1947 to circa 1980, the two had run close to each other, but from then on, wages were increasingly lagging behind productivity (Wolf 2017;25). Falling labor shares of national income has been an international trend since the 1970s (OECD 2015).

The core of the counter-offensive of capital was an embrace of de-industrialization in the North in favor of importing from, and outsourcing and out locating to low-wage, non-unionized countries and regions (like the US South), foreign investment, and a turn to financial operations, even by major former industrial corporations, like General Electric and General Motors. The coming of post-industrial societies did not mean more power to science and scientists, but to capital and capitalists. They could increasingly keep trade unions out of workplaces—only a third of OECD workers are now working under collective agreements (OECD 2019:20). They managed to make education, health care, and science sources of private capital accumulation, and they collected private riches beyond all comparison with other elite groups. From 1985 to 2010, the annual income of all employed Americans increased by 7% in real terms, but for a professor at the University of Chicago, the increase was 70%, and for a sample of big corporation CEOs between 502 and 825% (Hacker 2012:36). At a lower level of inequality, a similar pattern has been found in Sweden. Since 1950, the Swedish trade union congress (LO) has compared elite remunerations in Sweden in comparison with the average annual wages of industrial workers (Table 2).

**Table 2:** Industrial and post-industrial elite incomes in Sweden in 1950–2016, measured in average industrial workers’ wages

	1950	1980	2016
The business elite	26.1	9.1	88.3
Bureaucratic and professional elite	6.7	4.0	7.8
Elected political elite	4.3	2.9	3.8

Income includes salaries and other remuneration before tax. The business elite is a sample of CEOs of the biggest enterprises of a range of economic sectors. The bureaucratic and professional include top state appointees and top incumbents in universities and the media. The year of 1980 was the nadir of industrial inequality. Source: Landsorganisationen i Sverige, Makteliten .- toppnoterar igen, Stockholm, LO, 2018, pp. 21 and 12n

In terms of economic benefits and power, post-industrial societies have turned out business societies, above all. The skills permeated by the new economy are rather narrow.

## The two main drivers of current inequality

### Financialization

The first and the most direct force of the new inequality was financialization, “a pattern of accumulation in which profits accrue primarily through financial channels rather than through trade and commodity production” (Krippner 2005: 174), with “trade in securities on financial markets” (Godechot 2016:496) as its core activity.

It exploded with a remarkable simultaneity in the countries of the developed capitalist world in the 1980s, as manifested by one central aspect of it, stock market capitalization. Between 1870 and 1985, the median ratio of the domestic stock market value and GDP in seventeen capitalist countries (including the USA, the UK, France, Germany, and Japan) was stable, around a third of the domestic product. After 1985, it suddenly rose fivefold, from a low of 1/5 of GDP to level with GDP in 2000, and stayed at the new level (Kuvshinov and Zimmermann 2018:6). The USA, whose stock market is “comparable” in size to the other 16 countries combined (Kuvshinov and Zimmermann 2018:12), was at the center of the surge (Stein 2011; Krippner 2012). It spread rapidly, in the wake of the breakdown of the post-WWII international monetary system, fuelled by political deregulations of credit and capital markets, cuts of corporate and top income taxes, by innovative financial “products” (i.e., objects of trading and speculation), and cheered on by economic ideologies of deregulation and shareholder value. Competition between firms has “shifted from product markets to stock markets” (Erturk 2020:43 and ff). From 1980 to 2002, the share of financial sector profits in total US profits trebled from its previously stable share to a peak of 45%, staying around that level. The share of financial income in the profits of the non-financial sector of the economy rose from 15 to 42% in 2001, then descending to a third (Lin and Tomaskovic-Devey 2013:1284f). In 1990, financial assets amounted to half of world GDP, in 2015 to more than 400% of it (Lavinas 2018).

A large literature, both institutional (e.g., by OECD) and academic, has found that financialization has become a major driver of economic inequality (Assa 2012; Dünhaupt 2014; Godechot 2016; de Haan and Sturm 2017, Hermansen 2017). Calculations by the OECD have found that financialization, both in the form of increased stock market capitalization and increase of intermediated credit, has a polarizing effect on the income distribution, sinking disposable income growth for the bottom deciles and lifting it for the higher deciles, in particular the highest (Denk and Cournède 2015: 32 and 34). Among EU countries, operators in the financial sector make up a large proportion of the highest income earners, in the UK two thirds of the richest 0.1% (Denk 2015: fig. A2.18).

Sweden has experienced the largest increase of inequality in continental Western Europe under the new dynamic, which has included an extraordinary stock market capitalization expansion, from 9% of GDP in 1980 to 145% in 2017, proportionally the second largest in Europe (after Switzerland) (CEIC.data.com). The new centrality of the stock market constitutes a new system of corporate governance in Sweden, focused on “shareholder value,” typical of the Anglosphere, and different from that of France, Germany, and Japan. It is likely a major reason for Atkinson’s inclusion of Sweden among the countries with a U-turn of inequality (Cf. Sjöberg 2009; Roine and Waldenström 2012). Between 1991 and 2016, Swedish household income from work increased by 64%, from entrepreneurship by 62%, and from capital (interest, dividends, and, most importantly, capital gains from selling assets) by 229%. Income from capital is very unevenly distributed; 54% goes to the richest 1%, while the bottom 50% of the population receives 2% of it. In an econometric calculation, the Swedish Ministry of Finance shows that the whole inequality increase (and more) for 1995–2016 is due to the rising weight and concentration of capital income (Therborn 2020b).

The new inequality has been driven from the top by increasing income shares of the top 10%, top 1%, and top 0.1% in ascending order, virtually everywhere (Alvaredo et al. 2018: ch. 2.3). A significant accelerator has been the growth of stock options in corporate executive remuneration systems, powerfully stimulating stock market entrepreneurship and providing a vehicle for delinking CEO pay from that of other corporate employees. In 1980, an executive of a British corporation on the top FTSE (Financial Times/Stock Exchange share index) 100 list received a remuneration 18 times that of the median employee of the corporation; in 2018, it is 72 times (High Pay Centre 2016; 2019).

Financialization has decelerated since its heydays in 1999–2000 and 2006–2007, but financial wealth and income remain at a level not seen in Europe and North America since the Depression of the 1930s. It is a very powerful source of inequality since it refers to a kind of assets beyond the reach of the majority of the population. In the USA in 2013, the richest 1%% owned about half of all outstanding stock, financial securities, trust, business equity, and one third of non-home real estate. The top 10% held 85–90 of all financial investment assets and two thirds of all non-home real estate. Among middle-income households, their home accounts for three fourths of their assets, monetary savings for another 20%. Between 1983 and 2013, median net wealth fell among the poorest 60% of the US population, and median financial assets were close to zero among the poorest 40%. Between 1983 and 2013, the mean net worth of American households increased by 8369% for the richest 1%, by 634% for the next 19%, and by 6.6% for the middle 60% (Wolf 2017: 655f , 681, and 187, respectively). A significant aspect of financialization has been a post-1980 change of US pension (and other) systems, from “defined benefit” as a social right to “defined contribution” as an individual investor’s risk. In the USA, this change alone has increased wealth inequality by four Gini points (Wolf 2017: 355f).

The inequality effects of financialization, or of “financial integration,” variously measured, have been found consistently increasing across the Global South as well. The research overview by Zhang Juzheng et al. (2014: 40f) covers from East Asia to Latin America. Chile, under the military dictatorship, pioneered a replacement of its whole public pension system with one of the individual contributions to an array of private funds. In the second half of the 1990s, the World Bank pushed this system as a means to develop financial markets in Latin America and post-Communist Eastern Europe, with initial but short-lived success (World Bank 1994). The remnants of the financial market pension system were the main target of the large egalitarian demonstrations in Chile in late 2019, which have resulted in government promises of substantial reform.

### The digital technological revolution

Satellite TV, Internet, smartphones, mobile apps, robotics, and other products of the digital technological revolution have, in several ways, changed the world for the better, much better connected, much easier to handle in many everyday tasks, creating much more entertainment. However, in a distributive perspective, the overwhelming effect has been to increase inequality. The democratic potential of interactive social media, in comparison with unidirectional mass communication, while operating in some special political moments, like the Arab Spring in 2011, has largely been overwhelmed by corporate marketing by expert hackers and professional information-fakers.

The new digital technology has opened up a new field of capital accumulation, which in two to three decades has generated enormous wealth and monopolistic power for a few entrepreneurs and investors. Capitalism has acquired a new dynamic through the technical revolution. The background of its very core, Silicon Valley in California, is a dizzying brew of massive military investments in technological innovation, an excellent, private entrepreneurial university (Stanford), abundant private capital, and cultural openness to talents from everywhere, a venture driven by mixtures of military and civilian actors, academia and business, private and public capital, cosmopolitanism, and Americanism.^5^

Remarkably, this dynamic has not spawned any accelerating economic growth, neither in the USA nor in the world economy. On the contrary, world economic growth has stayed well below the 1950–1970 global trend line (World Bank Data), and in the USA well below the 1948-1970 growth rate (Gordon 2016:635). At least in part, this slowdown might be due to financialization and its corporate orientation of substituting “distribute and downsize” for “retaining and reinvest” [earnings] (Assa 2012). The economic impact of the digital revolution, so far, is thus mainly distributive.

The distributive impact of the digital revolution is most ostentatious in the fabulous personal wealth it has given its most prominent actors. Among the world’s ten richest persons, all men, as of August 24, 2020, according to Bloomberg Billionaire Index, they are seven (the top four) front figures of the digital technological change: Jeff Bezos (Amazon), Bill Gates (Microsoft), Mark Zuckerberg (Facebook), Elon Musk (SpaceX, Tesla), Steve Ballmer (Microsoft), Larry Page (Alphabet, Google), and Sergey Brin (Alphabet, Google). The individual rankings and exact fortune of these people are somewhat unstable, and Bloomberg’s Billionaire Index is a daily list, but the overall pattern is telling. Of the world’s twenty-five largest individual fortunes in August 2020, twelve have their origin in technologies that did not exist in 1980, only five were made in the pre-digital industry, four in retail trade, two in luxury goods provision, and two in private finance. A third of the largest fortunes (none of the top ten) are inherited or divorced.

The personal wealth of the technological frontrunners is to a large extent due to the connection of the new high tech with venture capital and financialization, first of all, with running the stock market. The five Big Tech companies, Apple, Microsoft, Amazon, Alphabet, and Facebook, account for 20% of all stock market capitalization in the USA. Together with their Chinese competitors like Alibaba and Tencent, they make up seven of the world’s eight most highly valued corporations. Their only rival is the Saudi oil company Aramco.^6^

The abundance of private capital available for digital enterprising has created a new path to high wealth—out of expansive loss-making. Ride-hailing Uber and music-streaming Spotify, for example, are world-wide corporations which so far have made losses every year of their existence but which have already made their founders into billionaires—by selling their shares of continuously rising market value.^7^

A major distributive effect of digital technology is its capacity to generate market power. Digitalized communication has made fast global diffusion and virtual monopolization possible, including attraction and influence, entertainment stars, product brands, and logistics firms. It is also making it possible to set up and control global value chains from afar. Global value chains control 80% of world trade, and a third is an intra-firm trade within multinational corporations (OECD 2016:60). A telling illustration of the new market power is the cost structure of Apple’s iPhones, analyzed by the business magazine *Forbes* and others. The latest seen by this writer concerns the iPhone10:

In percent of the retail price, the cost structure of iPhone10 is:

Components bought 37%

Manufacturing (wages of workers and profits of subcontractors) 2.5%

Profits for Apple designers and shareholders 60%.^8^

A profit margin of 60 to 70% seems to be general for smartphones, including Samsung’s (Techwalls 2019). The outsourced, subcontracted industrialization of the South, constantly under heavy cost pressure from powerful Northern customers, stymies the egalitarian potential of developed industrialization.

Finally, there is the asymmetrical power-enhancing effect of digital surveillance. The digital commandeering and monitoring of workers in Amazon’s warehouses, which so far have successfully fought off attempts at unionization and negotiated workers’ rights, have become international notoriety (Sanaito 2019; Guendelsberger 2019). Workers are regarded as competing with robots and get treated as disposables. The US economist Shoshana Zuboff (2019) sees the emergence of a wider control system, “surveillance capitalism.” Coalescence of applied science and finance has brought a post-industrial business society.

## Inequality, poverty, and the rise of the Global South

The resurrection of the South after centuries of subordination, in particular of China and India, has its world-historical significance primarily in geopolitics, as a historical shift of world power. However, it is also an epochal turning-point of global income patterns. International (population-weighted) inequality peaked around 1950 and started to decline in the second half of the 1980s, for the first time in two hundred years. At first, the bend of the curve was due exclusively to China (Milanovic 2005:142), but from 2000 international inequality is descending even excluding the ascent of China and India (Milanovic 2014). By 1950, both China and India had a lower GDP per capita than in the 1900s. With decolonization, both began to grow (Maddison 2001: 215, 265). However, this did not directly mean a catch up with the rich world, as the latter was growing vigorously after the war, and with lower fertility rates. India’s share of world GDP declined from 4.2% in 1950 to 3.1% in 1973, while China’s increased very slightly, from 4.5 to 4.6% (Maddison 2001:263). Only in the 1980s did China, India, and Indonesia begin their long and still unfinished approach to Western European GDP per capita, and since 2000, global inequality among households of the world is going down (Milanovic 2019:212, and 7, respectively).

### The enduring relativity of poverty

In the predominant, World Bank-orchestrated economic literature, the change of the South is mainly heralded as a historical reduction of human poverty. Is this the most meaningful interpretation? There is no doubt that an important reduction of extreme poverty has taken place—as has also happened with child mortality. The drastic reduction of extreme poverty in the world in the last 30 years is a major step in human progress. At the lowest level of World Bank-defined poverty, $1.90 a day in 2011 purchasing power parities, extreme poverty has decreased from 36% of the world population in 1990 to less than 9% in 2019 (World Bank 2020a). China—the most recent WB (World Bank) poverty rate, 0.5% in 2016 (World Bank 2020b: table 1.2.)—has driven world change. Between 1981 and 2005, the East Asian population in dire poverty declined by 755 million, while in the rest of the world numbers increased by 229 million (excl., Eastern Europe, Central Asia, the Middle East) (Ravaillon and Chen 2009: Table 1). There may be debatable measurement issues in gauging transitions from subsistence to monetarized economies, but anyway, this is a great achievement. Extreme poverty has become a regional problem of the South; in 2015, 56% of the poorest lived in sub-Saharan Africa and 33% in India (World Bank 2018: 2, 4).

However, poverty is a social concept, not a biological one, of survivability. It is intrinsically relational, referring to the disposal of resources lower than that of the median population. This state of lowliness may be defined at some more or less arbitrarily chosen level, as the original World Bank option for $1 a day in 1990—an approximation of the official poverty line of a selection of the world’s poorest countries, or in terms of the costs of a basket of basic consumer goods, as in the USA and Latin America. Alternatively, by the EU’s definition and by the Luxemburg Income Study, poverty may be defined as below a percentage of the median income of the population, 60% by the EU, 50% by the LIS (Luxemburg Income Study). While yielding different headcount numbers, both definitions are relational—defining poverty in relation to a given society and population, though the former is conventionally called “absolute” and the latter “relative.” The key architect of the World Bank’s dollar-defined poverty, Martin Ravaillon, has later made a “weak” concession to relativity by including a coefficient of mean national consumption in his calculations. The effect is rather dramatic: in 1981–2005 “absolute poverty” declined in East Asia and the Pacific from 78 to 17%, in “weakly relative” terms from 79 to 38%; South Asian “absolute” poverty headcount came down from 59 to 40% while increasing from 61.6 to 63.2% in relative terms; Latin American poverty changed level, 13% in “absolute” terms, reduced to 8%, while 52.5% going down to 45% when some consideration of average consumption is included (Ravaillon and Chen 2009:Tables 1-2).

The problem is not the true percentage of poverty. The problem is that poverty, unlike survival, is always relative, and after leaving one level of poverty, you may enter another one. In a world of growing intra-national inequality, this is most likely to happen to a large proportion of the population. The progress of living conditions which has taken place in recent decades is socially very important. However, it does not make up a historical turning-point, like the increase of inequality in the Global North and the decline of international and global household inequality. “Poverty” has not been abolished in the USA or anywhere in Europe, nor is relative poverty being abolished in China. Living conditions in China have improved tremendously in the past decades, but the human goalposts are moving with socio-economic development.

Therefore, the most significant social change in the Global South is not the reduction of some absolute poverty, but the shift of relative power and prosperity between the South and the North, and particularly between Asia and NATO-land, of Europe and North America. Explaining this shift is beyond the scope of this paper, but two aspects of the matrix of its dynamics may be noted. One is the rise in Asia of political and economic leadership with the knowledge and the post-colonial autonomy and self-confidence to make use of the opportunities opened up, by the new technologies, by the crisis of Northern industrialism, and by the new wave of capitalist globalization. The other is the enormous demographic weight of the major Asian countries once they start growing. The population of China is 4.3 times that of the USA, India’s population 15.55 times that of Germany, and Indonesia’s is 3.8 times the French population, Pakistan’s 2.9 times that of the UK.

### Is there a connection between declining global inequality and increasing intra-national inequality in the North?

The two inequality curves of Western intra-national inequality and the world’s international inequality changed direction at about the same time, in the second half of the 1980s. However, the triggers of the two were completely independent of each other. The rise of China, which, as we noticed above, first alone bent international inequality down, was due to a radical policy change in China’s reform and opening-up. The new Western inequality was Western capital’s response to the profit squeeze of the 1970s. The sharpening of international industrial competition, which played a major part in the fall of the profit rate in the USA and some European countries, like Sweden, did not come from China, but, above all, from Germany, Japan, and South Korea (Harrison and Bluestone 1988: ch. 2; Therborn 2020b). Evolutionary tendencies of relatively diminishing manufacturing employment were discernible in both the USA and Sweden before the 1970s crisis and had been part of Britain's long post-war decline.

For the OECD area as a whole, the most rapid acceleration of inequality occurred in 1985–1990 and 1995–2000, before the great global economic impact of China, from its WTO entry in 2001. Indeed the Chinese economic surge coincided with a deceleration of OECD intra-national inequality (OECD 2015: fig. 1.2), and one might add, with a restoration of US net profit rates to the golden level of 1945–1965 (Wolf 2017:26). China’s stunning export success in the first two decades of this century has greatly impacted US de-industrialization (Autor et al. 2016), and to a lesser extent, the rest of the OECD (Thevissen and van Vliet 2018) but increasing Northern inequality was fabricated in the Global North, domestically and through regional interaction, of financialization especially.

### Is industrial society relocating to the South?

De-industrialization in the Global North is coeval with industrialization in the South. What are the distributive prospects for the rise of Southern national income, particularly in Asia? Is a new, relatively egalitarian industrial society coming? In the 2000s, China became “the factory of the world,” a most impressive and unexpected achievement. However, neither China nor any country of the South is likely to become a fully industrial society. A decline of manufacturing employment, de-industrialization in Latin America and Korea began in the 1990s, in Malaysia after 2000 (UNCTAD 2016: table 3.2.). By the 2010s, manufacturing employment had stalled in China and India, while total industrial employment was being kept up by an increase in construction employment (ILO 2019) (Table 3). This means that industrial employment in the Global South will stop around the level of current Japanese and German de-industrialization (Table 1).

**Table 3:** Stalled industrialization: manufacturing and industrial employment in the Global South, 2010–2018 (percent of total employment)

	Manufacturing		Total industrial	
	2010	2018	2010	2018
Africa	8	7	13	13
China	22	22	28	29
India	11.4	11.6	23	25
Latin America	13	12	21	25
Southeast Asia	12	13.5	19	22

Source: ILO 2019, Employment in Industry, ILOSTAT 2019

Some further expansion of industrial employment in the South is not to be excluded, but it is very unlikely to reach even proximity to the peak of the most developed countries. Post-industrialism is becoming a global characteristic, a common foundation of an unequalizing world. Post-industrialism in the North means de-industrialization and de-standardization. In the South, it means stalled industrialization and persistent non-standardization or “informality.”

The halt of industrialization in the South means that egalitarian strivings will likely have no tailwind from comprehensive developed industrialism. In the short and medium terms, there are two effects of this stalling which need to be underlined. One concerns the future of the large populations still in subsistence or otherwise small-scale agriculture. In sub-Saharan Africa, peasants constitute 55% of the working population, in South Asia 43%, and in Southeast and East Asia, a third and a fourth, respectively (ILO 2019). For them, there will be no, or at most, a very narrow, industrial exit. Many of them risk being trapped in post-extreme poverty, in either rural or urban slums. The other problem is “informal employment,” basically work without contract and rights. It is a huge phenomenon. According to ILO (2018), it comprises 61% of the world’s working population. Recent economic development has had a very limited effect on it, if any (Table 4).

**Table 4:** Informal employment in some countries, 2009–2017 (percent of total)

Brazil	37
Egypt	51
India	85
Indonesia	73
Mexico	52
Pakistan	79
Philippines	70
Thailand	37
Vietnam	68

Source: ILO, Women and Men in the Informal Economy 2018

China is not included in the ILO (International Labour Organization) comparison, but informality is a significant, if minoritarian phenomenon on the Chinese labor market, e.g., among rural migrant workers without urban social benefits or a formal work contract.

Developed industrialism signified a formalization of the economy. Workers with an employment contract, and increasingly with a collective agreement, with workplace and social rights, replaced street vendors, family helpers, day laborers, and contractless workers in non-registered enterprises. Public social benefits unbound to employment may, to some extent, compensate for informal work status. However, the dualization of the labor market between formal and informal is a deep inegalitarian divide. The halted industrialization is a heavyweight force for the persistence of this divide.

## Convergent inequality patterns

China and the other rising big powers of Asia will not become industrial societies in the historical Euro-American sense. Furthermore, there is no “end of poverty” in sight, given the social-relational dimension of poverty. The tendency towards less inequality between peoples and territories of the globe is likely to continue unless disrupted by a catastrophic outcome of the COVID-19 pandemic. However, this global convergence includes another one, already visible, a development into similar inequality processes and patterns. *Exiting extreme poverty in Asia is coming to mean entering a post-agrarian, post-cum-pre-industrial society of inequality*. In fact, that was what happened to the poor of China and India simultaneously as they were lifted out of the worst misery. From 1978 to 2015, the share of national income going to the bottom half of the Chinese population declined from 27 to 15%, of the Indian from 23% in 1980 to 15% in 2015 (Alvaredo et al. 2018: fig. 2.1.1.e).

It should be noticed that the recent World Bank interest in “shared prosperity,” looking at the growth rate of the income or consumption of the poorest 40% of the population in relation to that of the total population, does not capture the inegalitarian and socially excluding effects of growing income gaps. For instance, because of the different starting levels of growth rates, the somewhat higher income growth of the bottom 40% of the Chinese population in 2013–2015 did not prevent the gap between their income and that of the population as a whole from increasing by 12% (from $5.55 to $6.25 a day) (World Bank 2018:63).

The OECD (2018, 2019) has put out grim overviews of the outlook of inequality and for the middle class in the Global North. It highlights the halt of upward mobility, the decline in the population share of income-earners between 75 and 200% of the median, the growing gap between the median income and the average of the top 10%, and more than one fifth of middle-income households spend more than they earn (Table 5). At present, it seems that the Global South is heading in the same direction. The drives of Southern inequality are beyond the scope of this paper, which no doubt include its own dynamic, e.g., related to rural-urban and agrarian-postagrarian transitions, but which already involve potent forces of financialization and digitalized capital accumulation.

**Table 5:** The bottom half and the top 10%. Income ratios in China, India, Europe, and the USA, 1980–2015. Before and after taxes and transfers (T&T)

	1980		2015	
	Before T&T	After T&T	Before T&T	After T&T
China	1:5	n.d.	1:14	n.d.
India	1:7	n.d.	1:20	n.d.
Europe	1:8	1:7	1:10	1:8
USA	1:8	1:6	1:20	1:12

Europe is all Europe excl. Russia, Ukraine, and Belarus. Sources: China (1978 and 2015) and India: World Inequality Database, www.wid.world/country; Europe and USA: Blanchet et al. 2019: 57f

In the South, the period was seen as an escape from poverty, but in the North, it is lived as an ousting from the relative egalitarianism of developed industrialism. The latter kind of society is unlikely to be reached by the much touted “emergent middle classes” of the South (Asian Development Bank 2011; Therborn 2020c).

The ongoing COVID-19 pandemic is sharpening the common intra-national inegalitarian tendencies of the world. At the same time, rather, it seems so far, strengthening the international convergence of the last three decades, as the epicenter of the pandemic has been in Western Europe and USA, and as East Asia has been the best managers of the challenge (hitherto). The most wealthy, the Big Tech corporations and their shareholders have been huge winners of the pandemic (Oxfam 2020; *New York Times* 21.8.2020), and the poorest have been the biggest losers, “informal” workers in the South and Northern “temporary” workers, ethnic minorities. Extreme poverty reduction is being reversed (Oxfam 2020¸ UN 2020; World Bank 2020b).

## Resistance and enlightenment

Post-industrial societies and financial capitalism have no tendentially pro-egalitarian class dialectic strengthening the poor and the exploited, unlike industrial capitalism. However, the inegalitarian denial of many people’s possibilities of realizing their capabilities will sooner or later generate resistance and protest movements. Increasing inequality is never a good recipe for social stability, though the time scales may be stretched.

However, the future of inequality will be made by its inherent dynamics, probable protests, and the always unpredictable historical contingencies. The world also includes an Egalitarian Enlightenment, not without influence, actual as well as potential. It has three main components. Most heavyweight is a plethora of organizations around the UN (United Nations), FAO (Food and Agriculture Organization), ILO, UNESCO (The United Nations Educational, Scientific and Cultural Organization), UNDP (United Nations Development Programme), UNICEF (United Nations International Children Emergency Fund), WHO (World Health Organization), and others, which are all committed and institutionally empowered to a humane egalitarian agenda. They have made important contributions to poverty alleviation and the reduction of vital and existential inequality. Their economic clout has always been limited, though. Another component is an embryonic global civil society of organized concerned citizens. In the field of inequality concern, Oxfam is perhaps the most important example. Thirdly, and most recent, is a shift among economists, supported by other social scientists from more egalitarian traditions. Promoted by the work of Thomas Piketty and his associates, and highlighted by crises and protests, from the crash of 2008 to the ongoing COVID-19 pandemic, inequality has become a hot topic. Inequality studies are set up as centers, institutes, or programs in elite universities from Paris and London to Harvard, Stanford, and Berkeley and spreading across continents. The pioneer Amartya Sen and four recent Nobel Laureates of economics are involved, Joseph Stiglitz, Angus Deaton, Abhijit Banerjee, and Esther Duflo. The new Managing Director of the IMF (International Monetary Fund), once the spearhead of anti-egalitarianism, is issuing a call to “Reduce Inequality to Create Opportunity” (Georgieva 2020). The founder and Director of the World Economic Forum, advocating a “Great Reset” of a “better capitalism” (Schwab and Malleret 2020), and the editors of the London *Financial Times* (April 3, 2020, www.ft.com) have also recently come out as egalitarians. While the bourgeoisie has intermittently harbored a few socially concerning exceptions, within the “dismal” discipline of economics, this phalanx of front-rank egalitarians seems to be yet another historical turn.

The powerful twenty-first century dynamics of inequality, North and South, are not likely to unfold without critical questioning.

## Data Availability

N/A.

## Abbreviations

COVID-19: Coronavirus disease 2019
FAO: Food and Agriculture Organization
FTSE: Financial Times/Stock Exchange share index
ILO: International Labour Organization
IMF: International Monetary Fund
LIS: Luxemburg Income Study
OECD: Organization for Economic Co-operation and Development
OPEC: Organization of the Petroleum Exporting Countries
T&T: Taxes and transfers
UN: United Nations
UNDP: United Nations Development Programme
UNESCO: The United Nations Educational, Scientific and Cultural Organization
UNICEF: United Nations International Children Emergency Fund
WB: World Bank
WHO: World Health Organization
WWII: World War II

## Footnotes

1 See further Therborn (2013).

2 Atkinson and Piketty (2010): national chs. and ch. 13, who see the second pattern more as an L.

3 See further Therborn (2004:ch. 5.)

4 For Swedish sources, see further Therborn (2020b).

5 See further, e.g., O’Hara (2019).

6 New York Times 21.8.20, pp. 1, 8; Wikipedia.org. 24.8.20; Statista.com 19.8.20.

7 On Spotify, a Swedish-originated company, see Pettersson and Wisterberg (2019:149-205).

8 Tricontinental (2019).
